# Modeling airway persistent infection of *Moraxella catarrhalis* and nontypeable *Haemophilus influenzae* by using human *in vitro* models

**DOI:** 10.3389/fcimb.2024.1397940

**Published:** 2024-05-01

**Authors:** Andrea Ariolli, Martina Canè, Martina Di Fede, Simona Tavarini, Anna Rita Taddei, Kevin Pete Buno, Isabel Delany, Silvia Rossi Paccani, Alfredo Pezzicoli

**Affiliations:** ^1^ Department for Innovation in Biological, Agro-Food and Forest Systems, University of Tuscia, Viterbo, Italy; ^2^ Department of Biology, University of Naples Federico II, Napoli, Italy; ^3^ GlaxoSmithKline Vaccines s.r.l., Preclinical R&D, Siena, Italy; ^4^ Great Equipment Center-Section of Electron Microscopy, University of Tuscia, Viterbo, Italy

**Keywords:** AECOPD, airways, *in vitro* models, microscopy, replacement, CIVM

## Abstract

Non-typeable *Haemophilus influenzae* (NTHi) and *Moraxella catarrhalis* (Mcat) are two common respiratory tract pathogens often associated with acute exacerbations in Chronic Obstructive Pulmonary Disease (COPD) as well as with otitis media (OM) in children. Although there is evidence that these pathogens can adopt persistence mechanisms such as biofilm formation, the precise means through which they contribute to disease severity and chronicity remains incompletely understood, posing challenges for their effective eradication. The identification of potential vaccine candidates frequently entails the characterization of the host-pathogen interplay *in vitro* even though this approach is limited by the fact that conventional models do not permit long term bacterial infections. In the present work, by using air-liquid-interface (ALI) human airway *in vitro* models, we aimed to recreate COPD-related persistent bacterial infections. In particular, we explored an alternative use of the ALI system consisting in the assembly of an inverted epithelium grown on the basal part of a transwell membrane with the aim to enable the functionality of natural defense mechanisms such as mucociliary clearance and cellular extrusion that are usually hampered during conventional ALI infection experiments. The inversion of the epithelium did not affect tissue differentiation and considerably delayed NTHi or Mcat infection progression, allowing one to monitor host-pathogen interactions for up to three weeks. Notably, the use of these models, coupled with confocal and transmission electron microscopy, revealed unique features associated with NTHi and Mcat infection, highlighting persistence strategies including the formation of intracellular bacterial communities (IBCs) and surface-associated biofilm-like structures. Overall, this study demonstrates the possibility to perform long term host-pathogen investigations *in vitro* with the aim to define persistence mechanisms adopted by respiratory pathogens and individuate potential new vaccine targets.

## 1 Introduction

The human respiratory system is a complex organ that provides a critical line of defense against inhaled pathogens and represents a niche for bacterial colonization (Kumpitsch et al., 2019). Along almost its entire length, from the nasal cavity to the bronchi, the respiratory mucosa is characterized by the presence of a pseudostratified columnar ciliated epithelium set on the underlying basement membrane. Differently, the bronchiolar cavity is lined by a simple columnar to cuboidal epithelium, while alveoli are characterized by a thin squamous epithelium that facilitates gas exchange. Depending on the airway region, the respiratory epithelium displays a specific cell type composition and distribution that is related to its function (Morgenroth and Ebsen, 2008; Ball et al., 2022; Kia’i and Bajaj, 2022). Basal cells, representing approximately 6-31% of the human airway epithelium, are undifferentiated cuboidal-shaped stem cells that carry out epithelial self-renewal function (Boers et al., 1998). Ciliated and non-ciliated secretory epithelial cells line most of the respiratory tract (from the upper airways to the bronchi) playing a fundamental role in lung defense from pathogens and inhaled particles. Indeed, ciliated cells act in synergy with mucus-producing goblet cells to promote the mucociliary escalator (or mucociliary clearance, MCC), consisting in the transport out of the lungs of mucus-entrapped foreign particles (Bustamante-Marin and Ostrowski, 2017). Goblet cells represent the most common secretory cell type in the tracheobronchial district (Barron et al., 2021; Davis and Wypych, 2021) followed by club cells that produce a variety of products, including uteroglobin and surfactant that respectively regulate inflammation and protect the bronchiole lining (Reynolds and Malkinson, 2010).

Advanced *in vitro* airway models are laboratory-grown cell-based systems designed to provide a micro physiological surrogate of specific districts of the respiratory tract. These models auspiciously represent an alternative to animal testing for new therapeutics safety and efficacy evaluations and can be employed to investigate many diverse biological processes and/or related pathological conditions, such as tissue differentiation (Prytherch et al., 2011), tissue regeneration (Puchelle et al., 2006; Chakraborty et al., 2021), inflammation, infection and chronic disease (Miller and Spence, 2017; Yonker et al., 2017; Osei et al., 2020). Among the most popular systems used to study the host-pathogen interplay occurring in the human airways stands the Air-Liquid Interface (ALI) transwell model that allows one to develop highly differentiated healthy or diseased models of the human respiratory system (Hasan et al., 2018; Baldassi et al., 2021). ALI models are assembled by culturing epithelial cells on a porous membrane that divides the inner chamber from the culture well of the holding plate. Upon confluence, the cells are then airlifted, and the use of specific media facilitates tissue maturation into a fully differentiated respiratory epithelium. To enhance the tissue physiology, the complexity of the model can be improved by co-culturing the respiratory epithelial cells with extracellular matrix (ECM)-embedded fibroblasts located on the basal side of the membrane. In fact, the interaction between the pseudostratified epithelium and its basal microenvironment has been reported to have a role in multiple aspects including epithelial cell function, spatial distribution, and differentiation (Kobayashi et al., 2006; Pageau et al., 2011; Steinke et al., 2014). The resulting tissue exhibits *in vivo*-like features including the presence of multiple cell types and the production of substantial amounts of mucus along with sustained ciliary beating, which together represent a major component of the innate defense at the airway mucosal interface. Indeed the presence of a mucus layer, besides being an essential factor for a functional MCC, is particularly important because it prevents direct accessibility to the epithelium where pathogens can use cellular receptors to adhere to the tissue (Caldara et al., 2012).

When referring to pathogenic bacterial infection studies, one limitation of this and other models is that the well is physically confined, leading to the accumulation of growing bacteria during time that causes the disruption of the epithelium within few hours/days. Recent reports (Yonker et al., 2017; Zaderer et al., 2019) demonstrated the feasibility to culture a respiratory epithelium on the downward face of the transwell membrane (inverted configuration) allowing to conduct studies on immune cell transmigration. Besides the possibility to reproduce aspects of the immune system at the respiratory barrier the inverted ALI epithelial configuration opens new possibilities to look at the bacterial-host interaction in an unconfined space.

Non-typeable *Haemophilus influenzae* (NTHi) and *Moraxella catarrhalis* (Mcat) are two respiratory tract pathogens frequently associated with OM and acute exacerbation episodes in COPD patients (AECOPD) (Blakeway et al., 2017; Bakaletz and Novotny, 2018; Mayhew et al., 2018; Weeks et al., 2021). Extensive investigation on multiple aspects of NTHi (Ahearn et al., 2017; Sriram et al., 2018; Zhang et al., 2020; Weeks et al., 2021) and Mcat (Goldstein et al., 2009; Murphy and Parameswaran, 2009; Blakeway et al., 2017) pathogenesis provided insights into their virulence mechanisms, aiming to define strategies to combat or reduce their health-related burden. Several studies reported the ability of these gram-negative bacteria to adopt persistence mechanisms such as biofilm formation and intracellular survival (Slevogt et al., 2007; Clementi and Murphy, 2011; Bair and Campagnari, 2019). However, the way these pathogens contribute to disease severity and chronicity in the airways are still not fully elucidated (Weeks et al., 2021). Biofilms are complex communities of microorganisms embedded within a self-produced matrix that protects cells from the external environment, bolstering bacterial resistance to treatments and immune defenses (Flemming and Wingender, 2010), and contributing to pathogen persistence and disease chronicity (Bjarnsholt, 2013). Intracellular bacterial communities (IBCs) are formed by invading bacteria that upon entry into epithelial cells rapidly divide to form an intracellular biofilm-like niche protected from innate defenses and antibiotics (Mirzaei et al., 2020). Supporting evidence indicate the formation of IBCs by NTHi during chronic (Hall-Stoodley et al., 2006; Thornton et al., 2011) and experimental OM (Hardison et al., 2018) whereas a less clear indication of NTHi extracellular biofilm formation in humans exists even though it is widely recognized as a persistence mechanism adopted by the pathogen *in vivo* (Weeks et al., 2021). Similarly, Mcat has been reported to form biofilms *in vivo* (Armbruster et al., 2010; Perez et al., 2014) and to invade host cells (Slevogt et al., 2007) as strategies to prevent clearance.

In the present work, we considered the use of conventional and inverted human airway ALI systems to model respiratory infections caused by NTHi and Mcat. In particular, we discovered that progression of infection of both pathogens is slowed down in inverted models in comparison to standard ALI cultures, possibly due to functional MCC, cell extrusion and/or other mechanisms. Moreover, by applying a microscopy-based approach, we found that both NTHi and Mcat are capable of invading epithelial cells and forming IBCs and/or large biofilm-like structures intraepithelially or on the apical side of the epithelium, supporting the evidence that both pathogens can adopt resistance strategies that likely result in incomplete clearance during human infection.

## 2 Materials and methods

### 2.1 Airway epithelial models

Primary human airway epithelial cells isolated from the upper airways (hAEC) of diseased (COPD) subjects were purchased from Epithelix. Cells were expanded in 75 cm^2^ flasks by using human Airway Epithelial Cell Culture medium (hAEC; Epithelix) supplemented with Primocin (Invivogen), at 37°C in 5% CO_2_ until approximately 80% confluence and used between passage 4 and 6. Standard and inverted models were cultivated according to a similar protocol, with minor modifications required for the inverted as already described (Yonker et al., 2017). For both configurations, 6.5 mm transwell with 0.4 µm pores were used (Corning product #3470) and the inner or outer side of the insert membranes were coated with a Collagen Type I solution from rat tail (REFC3867-1V, Sigma-Aldrich) prior to epithelial cell seeding. To produce conventional ALI models, ~0.5 x 10^5^ cells were applied in the apical chamber of the inserts whereas for inverted models transwells were flipped and ~1 x 10^5^ cells were seeded on the membrane; after one hour of incubation at 37°C to allow adhesion, unbound cells were carefully aspirated, and the inserts were returned to the receiving wells containing 600 µl of medium. Cells were cultivated under submerged conditions for 3-5 days to allow the formation of a confluent monolayer. Subsequently, to induce cellular differentiation, the models were cultured at the Air-Liquid-Interface with complete Pneumacult-ALI medium (StemCell Technology) changing medium every 2 days. After two weeks, a 25% Matrigel (Corning) layer containing ~3 x 10^3^ human fibroblasts (Normal Human Lung Fibroblasts or IMR90, ATCC) was added on the underside (standard configuration) or upside (inverted configuration) of the insert membrane and Pneumacult was replaced with SmallAir medium (Epithelix). Cellular models were cultured in ALI for at least 28 days prior to their use to ensure the full differentiation of the tissue. Cell polarity and TJ barrier function were verified by transepithelial electrical resistance (TEER) measurements using an epithelial volt ohmmeter (EVOM2; World Precision Instruments). TEER variation of standard and inverted models was monitored during the differentiation process and the experimental infections. Measurements were taken after placing the models in submerged conditions by adding culture medium to the basal or apical chamber of the inverted (600 μL) and standard (300 μL) models, respectively. TEER measurement during infection experiments were performed at end-points only to avoid any effect on the progression of infection. Differentiation and polarization were evaluated by confocal scanning laser microscopy (Zeiss LSM 710) through the labelling of specific markers for the presence of cilia, tight junctions, goblet cells, basal cells, and club cells.

### 2.2 Bacterial culture and model infection

NTHi strain Fi176 (kindly provided by Richard Moxon and Derek Hood, Oxford University, UK), isolated from a child with otitis media, and *Moraxella catarrhalis* strain 415, isolated during the prospective, observational cohort AERIS study (Wilkinson et al., 2017) (NCT01360398) were used in this work.

All the strains used were routinely reconstituted from frozen stock cultures, plated on PoliVitex chocolate agar (bioMérieux), and incubated overnight at 37°C with 5% CO_2_. For infection studies, Brain-heart infusion (BHI) was used as growth medium at 37°C, 180 rpm with 5% CO_2_. For NTHi growth, BHI was supplemented with 5 μg/ml hemin and 2 μg/ml of nicotinamide adenine dinucleotide (NAD, Sigma). Bacteria concentration was determined by both measuring the absorbance at 600 nm (OD_600_) and colony forming units (CFU) by dilution plating. 24 hours before the infection the culture medium was replaced with Small Air medium (Epithelix) without antibiotics. On the day of the infection, 100 µl of bacterial suspension (corresponding approximately to ~3 × 10^8^ CFU/mL for Mcat AERIS 415 and ~1 × 10^9^ CFU/ml for NTHi Fi176) or medium-only control were added to the apical (standard) or bottom (inverted) side of the transwells and incubated for 1 h at 37°C. Subsequently, epithelia were washed three times with D-PBS to remove non-adherent bacteria and models were maintained in Small Air medium until the end of the experiment without washing the epithelial surface again.

### 2.3 Laser scanning confocal microscopy

Cellular models were washed three times with PBS and fixed with 4% formaldehyde (FA), except for mucin staining, where a methanol-acetone (1:1) fixation protocol was used. Membranes were cut with a scalpel and processed in a 48-well plate. Samples were permeabilized with a 1% saponin (Sigma), 0.1% Triton X-100 (Sigma) solution in PBS for 30 minutes and blocked with 3% bovine serum albumin (Sigma), 0.1% Triton X-100 in PBS for 45 minutes. Incubation with primary antibodies was performed for 1 h at room temperature; then, samples were washed in blocking solution and treated for 30 minutes with the appropriate Alexa Fluor-conjugated secondary antibody (Thermo Fisher). Membranes were mounted on microscopy slides using ProLong gold antifade reagent (Thermo Fisher) and analyzed by confocal microscopy using either a Zeiss LSM710 confocal microscope or Opera Phenix HCS Plus (PerkinElmer). Image analysis was performed using Harmony HCI (PerkinElmer) or Vision4D (Arivis) software.

Images obtained by acquiring the whole transwell membrane with Opera Phenix HCS System were analyzed with Harmony HCI software with the aim of 1) comparing the quantity of ciliated and goblet cells of models grown in standard or inverted configuration; 2) comparing the bacterial load of standard or inverted models challenged with NTHi Fi176. A dedicated analysis pipeline was developed for each workpackage (**Supplementary Figures 1, 2**). Ciliated and goblet cell abundance was estimated through virtual segmentation of the fluorescence signal related to ß-tubulin IV or MUC5CAC, respectively, whereas bacteria were quantified by segmentation of the fluorescence signal associated with NTHi-targeting antibodies. Z-stacks were acquired with a 40x water objective and segmentation of fluorescence was performed on maximum projections. Quantification of the different markers was done by calculating the sum of the fluorescence area (µm^2^) of 144 fields divided by the sum of the area of the fields in order to obtain a percentage for each marker. Antibodies and dyes used in this study are summarized in **Supplementary Table 1**.

### 2.4 Electron microscopy

Protocols were adapted from Hammerschmidt et al. (2005) with little modifications. Membranes were pre-fixed in a cacodylate sucrose buffer (0.1 M Sodium Cacodylate, 0.09 M sucrose, 0.01 M CaCl_2_, 0.01 M MgCl_2_ and 0.075% Ruthenium Red, pH of 6.9) containing 2% paraformaldehyde (PFA), 2.5% glutaraldehyde (GA) and 1.55% L-lysine acetate (all reagents from Sigma) for 20 minutes at 4°C. Then samples were soaked in the same solution without L-lysine acetate overnight at 4°C (second fixation). For transmission electron microscopy (TEM), samples were post-fixed in 1% OsO_4_ (Electron Microscopy Science) and 0.075% Ruthenium Red in 0.1 M Cacodylate buffer, pH 6.9, 1 h at 4°C, rinsed in bidistilled water for 15 minutes at 4°C and dehydrated in a graded series of alcohol concentrations prior to embedding in LR White resin (Agar Scientific) at 50°C for 36 hours. Ultrathin sections (60-80 nm) were obtained using a Reichert-Jung ultracut E ultramicrotome and stained with uranyl acetate prior to examination with a JEOL 1200 EX II transmission electron microscope at an electron accelerating voltage of 100 kV equipped with an Olympus SIS Veleta CCD camera and the iTEM software for image acquisition and processing. For scanning electron microscopy (SEM) observations, samples were fixed and dehydrated as mentioned above. Samples were dehydrated by using the method of liquid CO_2_ in a Critical Point Dryer (Balzer Union CPD 020), coated with gold in a Sputter Coater (Balzer Union MD 010) and examined using a JEOL JSM 6010 LA at 5-15 kV voltage acceleration. For Immuno-Gold labelling assays, membranes were fixed with 4% PFA and 0.02% GA in 0.1 M Phosphate Buffer (PB), pH 7.4, for 2 hours at 4°C, then rinsed with 0.1 M PB and incubated in a 0.2 M sucrose solution overnight at 4°C. Subsequently, samples were dehydrated and embedded in LR White resin as described above. Ultrathin sections were processed according to a two-step blocking procedure (low and high molecular weight, respectively) using firstly Glycine 0.05 M in PBS for 15 minutes and then Aurion blocking solution for 30 minutes. Samples were washed two times with incubation buffer and treated with the appropriate primary antibody for 3 hours at RT or overnight at 4°C. Then, after being washed 6 times with incubation buffer, samples were treated with the appropriate secondary antibody for 2 hours and finally washed 6 times with PBS.

### 2.5 Flow cytometry

For the flow cytometry analysis, infected or mock-infected airway epithelial models were processed according to previously defined infection time-points, indicated in the figure legends. Epithelia were detached and dissociated into single cell suspension according to the following protocol. D-PBS w/o calcium and magnesium was added on the apical side of the models and cells were incubated at 37°C for 5 minutes, then rinsed three additional times with the same buffer. Culture medium was removed and 300 µl of TrypLE Select Enzyme 10X (Thermo Fisher) were added to the apical and the basal side of each well; cells were incubated at 37°C for 5-10 minutes and manually agitated. Cell aggregates were dissociated into single cell suspensions by gently pipetting. Cell suspensions were transferred into 2 ml tubes containing 700 µl of D-PBS and pelleted at 0.3 x g for 5 minutes. Cell pellets were resuspended in 300 µl of LIVE/DEAD Fixable Aqua Dead Cell (Thermo Fisher) staining solution (1:600 in PBS) and incubated at room temperature for 10 minutes. Samples were resuspended in 4% FA and incubated on ice for 20 minutes, then washed with PBS and resuspended in PBS 1% BSA before analysis. Data were collected using a BD FACS CANTO II (BD Bioscience) and the analysis was performed using Flow-Jo Software (v.8.6, TreeStar Inc). The first two gates were drawn to identify the cell population of interest by using a dot plot forward versus side scatter area (FSC-A vs SSC-A). The second gate was drawn on single cells using a dot plot side scatter width versus side scatter area (SSC-W vs SSC-A) to exclude cell aggregates. Then, live and dead cells were identified with a dot plot SSC-A vs Live/Dead Aqua-A. The mean percentages of cell death and debris were analyzed according to Welch and Brown-Forsythe ANOVA followed by Dunnet’s T3 post-hoc multiple comparison test using the GraphPad Prism (software version 9.5.1).

## 3 Results

### 3.1 Epithelial differentiation of inverted and standard airway models is comparable

**Figure 1:**
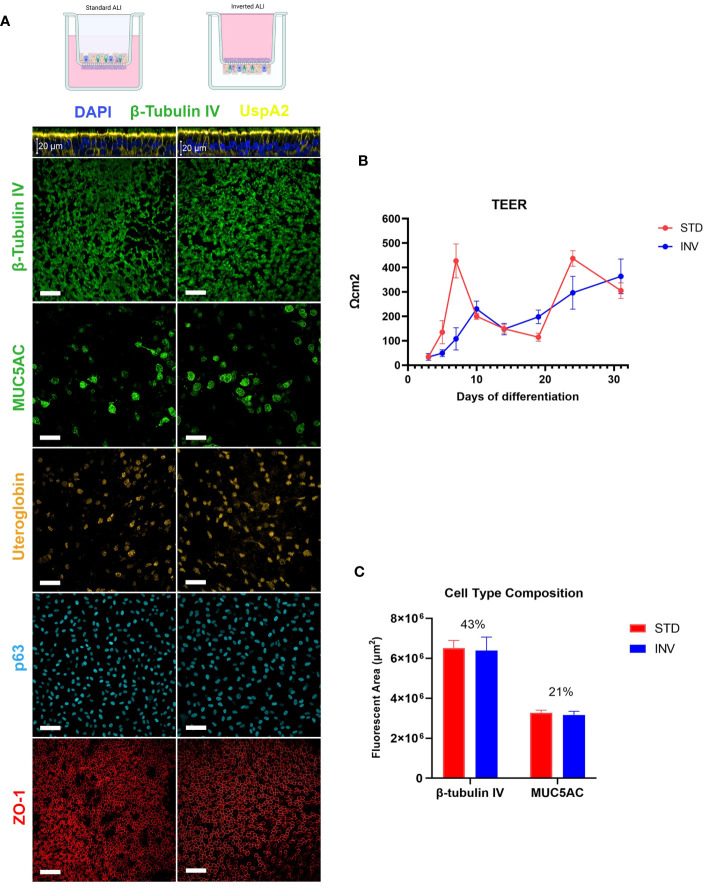
Characterization of ALI respiratory models. **(A)** LSCM analysis of epithelial differentiation: cilia (β-tubulin IV), mucus (MUC5AC), club cells (Uteroglobin), basal cells (p63) and Zonula Occludens 1 (ZO-1) were present in both standard and inverted models (white bars = 50µm). **(B)** Trans-epithelial electrical resistance measurement during epithelial differentiation. Results are expressed as mean values plus standard deviation calculated on 25 replicates for each configuration. **(C)** Evaluation of ciliated and goblet cells relative proportion based on the quantification of the fluorescent area related to β-tubulin IV and MUC5AC, respectively. Bars represent mean values plus standard deviation calculated on three replicates from three independent experiments (144 fields were analyzed for each sample).

Classical ALI models consist of cells grown in the inner chamber of a transwell system where spatial confinement does not allow for the expulsion of bacteria that accumulate during infection. To overcome this limitation, we set up an inverted ALI model where the epithelium was assembled on the bottom face of the transwell porous membrane, potentially enabling the discharge of proliferating bacteria and infected cells. In order to exclude the impact of any potential difference between standard and inverted models on the infection phenotype, we first wanted to verify that models grown in different configurations had the same characteristics. Therefore, after assembling conventional and inverted airway models, phenotypical and functional characteristics of the epithelia were evaluated. By monitoring Trans-Epithelial Electrical Resistance (TEER) we found that both types of models could form a functional epithelial barrier following air exposure. Even though data indicated that, during the first 7 days of culture, TEER values were higher in standard models, the overall trend between the two configuration was comparable and the values finally overlapped after 31 days of culture (**Figure 1B**). Consistently with this observation, Zonula Occludens 1 (ZO-1) protein, which is part of the multiprotein junctional complex responsible for apical barrier function, was found properly delineating inter-cellular edges at the apical interface (**Figure 1A**). Laser Scanning Confocal Microscopy (LSCM) analysis highlighted the development of a pseudo-stratified tissue as well as the presence of specific differentiation markers indicating the existence of ciliated (β-tubulin IV), goblet (MUC5AC), club (uteroglobin), and basal cells (p63) in both configurations (**Figure 1A**). In particular, cilia were evenly distributed along the apical surface whereas goblet and club cells were found less frequently among ciliated cells, populating some areas with higher density. Basal cells uniformly lined the bottom region of the epithelium. Both types of models exhibited sustained ciliary beating, mucus production and regenerative capacity after damage (data not shown). To ensure that MCC could be equally supported by standard and inverted models, the proportion of ciliated and goblet cells in each configuration was estimated by image analysis (**Figure 1C**). Results indicate that cilia cover approximately 42–44% of the analyzed area both in standard and inverted models, representing the most abundant cell type. The analysis appears in accordance with values reported for the human airways, according to which ciliated cells are the predominant type, representing 50–80% of epithelial cells, depending on the airway region (Crystal et al., 2008; Yaghi and Dolovich, 2016). Goblet cells are the most common secretory cells in the tracheobronchial epithelium with their abundance varying from 25% in the upper respiratory tract to progressive lower values in small airways where club cells represent the most abundant secretory cell type (Rogers, 2003). Also in this case, regardless from the configuration, goblet and club cells were equally distributed over the epithelium, representing approximately 20% of the total area.

### 3.2 Mcat and NTHi infection is attenuated on inverted airway models

**Figure 2:**
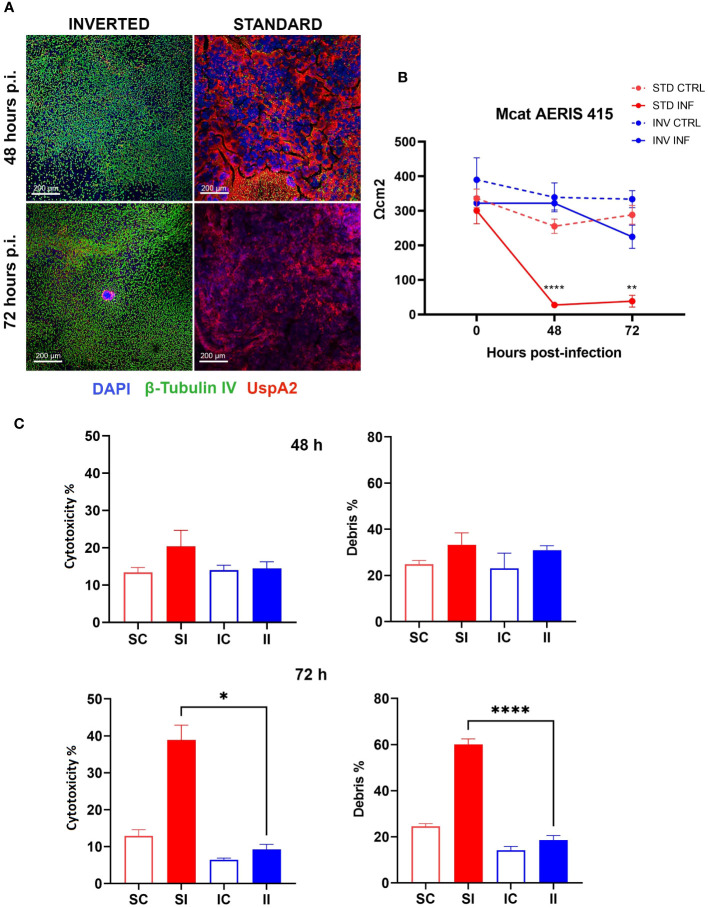
Inverted respiratory models better tolerate Mcat infection. **(A)** LSCM analysis of standard and inverted models 48 and 72 hours after Mcat AERIS 415 inoculum. Unlike inverted models, standard models are completely covered by bacteria (Green are cilia, red is Mcat, Blue is DNA; white bars = 200 µm). **(B)** TEER measurement during 72 hours of Mcat infection. Results are expressed as mean values (plus standard deviation) of four replicates for each condition. **(C)** Cytotoxicity and debris analysis of standard and inverted models 48 and 72 hours after Mcat challenge. Bars represent mean value (plus standard deviation) calculated on three replicates for each condition (SC, Standard Control; IC, Inverted Control; SI, Standard Infected; II, Inverted Infected); data is representative of three independent experiments. Data were analyzed according to Welch and Brown-Forsythe ANOVA followed by a Dunnet’s T3 post-hoc test. Statistical significance for the difference between the means of SI and II is reported (*p < 0.05; **p < 0.005; ****p < 0.0001).

**Figure 3:**
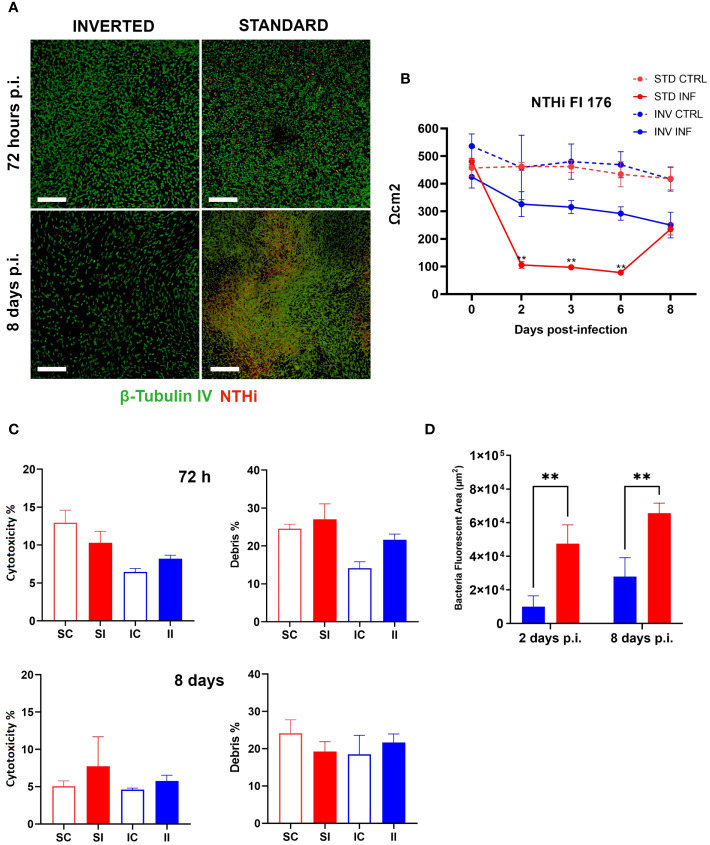
NTHi infection is better controlled on inverted models. **(A)** LSCM analysis of standard and inverted models 72 hours and 8 days after NTHi infection (Green are cilia, red is NTHi; white bars = 200 µm). **(B)** TEER measurement during 8 days of NTHi Fi176 infection. Results are expressed as mean values [plus standard deviation] of four replicates for each condition. **(C)** Cytotoxicity and debris analysis of standard and inverted models 72 hours and 8 days after NTHi challenge. Bars represent mean value (plus standard deviation) calculated on three replicates for each condition (SC, Standard Control; IC, Inverted Control; SI, Standard Infected; II, Inverted Infected); data is representative of three independent experiments. **(D)** Quantification of bacteria colonizing standard or inverted models 2 and 8 days after infection. The total area associated with bacterial fluorescence from 25 random fields was quantified with the Harmony software. Bars represent mean values (plus standard deviation) calculated on three replicates. Data were analyzed according to Welch and Brown-Forsythe ANOVA followed by a Dunnet’s T3 post-hoc test. Statistical significance for the difference between the means is reported (**p < 0.005).

As previously stated, we hypothesized that by inverting the epithelial configuration, we could exploit the full capability of the epithelium to counteract bacterial infection. To test this possibility, we challenged standard and inverted models with NTHi Fi176 or Mcat AERIS 415 strains and followed the infection for 8 and 3 days, respectively. The epithelial surface of inverted models was exposed by turning transwells upside down and applying bacterial suspensions for an hour before washing to remove unbound bacteria. We found that the extent of bacterial adhesion after 1 h of infection with Mcat or NTHi was comparable for both configurations (data not shown), ensuring an equivalent initial bacterial load for standard and inverted tissues. To evaluate the effect of the infection on the integrity of the epithelial barrier, TEER was monitored during the course of the experiment (**Figures 2B**, **3B**). Results indicated that, for both pathogens, TEER values of infected inverted models were significantly higher respect to standard models. In particular, 48 hours after Mcat inoculum, a remarkable TEER decrease was observed in standard models, suggesting failure of epithelial barrier function whereas equivalently inoculated inverted tissues maintained higher TEER values, comparable to uninfected tissues (**Figure 2B**). Similarly, TEER readings of NTHi-infected samples decreased earlier in standard models (48 hours) respect to inverted samples, where the reduction was less pronounced and constant during the entire infection time-course (**Figure 3B**). Unexpectedly, 8 days after infection, TEER of infected standard models increased and the values of the two configurations converged, suggesting a partial recovery of the barrier function. LSCM analysis conducted on Mcat infected samples showed that after 48 and 72 hours the difference in bacterial colonization between standard and inverted models was quite noticeable (**Figure 2A**). Indeed, at 48 hours standard models challenged with Mcat appeared widely covered by large bacterial aggregates while in inverted models the pathogen was mainly found as single cells or small bacterial aggregates. At 72 hours post-infection, Mcat completely invaded standard models while, in inverted tissues, macro-colonies started to appear on the epithelium, which still maintained an intact tissue architecture. Analysis conducted on NTHi infected samples provided results in accordance with TEER variations. Indeed, despite NTHi did not display a tissue invasiveness comparable to that of Mcat (**Figure 3A**).

In order to assess the impact of bacterial infection on cell viability, standard and inverted models were dissociated into single cell suspensions and analyzed by flow cytometry after staining with LIVE/DEAD cell stain (**Supplementary Table 1**). **Figures 2C**, **3C** summarize the results obtained for Mcat and NTHi, respectively. In particular, data revealed a higher cytotoxicity induced by Mcat infecting standard tissues respect to inverted models at 72 hours post-infection (39 vs 9%, p < 0.05). Notably, this analysis takes under consideration only cells that still exhibit a shape and morphology similar to those of normal cells but doesn’t include cells that have undergone degradative processes following cell death. Therefore, we analyzed the debris content in each sample to discover that, although no significant differences were observed at 48 hours post-infection, standard models infected with Mcat displayed a significantly higher amount of debris at 72 hours post-infection respect to inverted samples (60 vs 19%, p < 0.0001). Interestingly, the same analysis performed with models infected with NTHi Fi176 strain did not highlight differences in the cell cytotoxicity rate between inverted and standard tissues (**Figure 3C**). Accordingly, the analysis of cellular debris indicated that the infection of NTHi did not induce a significant increase of cellular debris in standard infected models. Nevertheless, the difference in the level of bacterial colonization between standard and inverted models was evident. To highlight this difference, we developed an image-based approach able to quantify bacteria at different time points (**Supplementary Figure 2**). As expected, the analysis showed that NTHi colonization was lower in inverted respect to standard models (**Figure 3D**).

Interestingly, LSCM analysis of the epithelia at 8 days post-infection highlighted differences in the cellular architecture between standard and inverted tissues. In particular, large portions of the inverted epithelium exhibited reduced thickness and fewer ciliated cells in comparison with the standard (**Supplementary Figure 3**), possibly a consequence of cell extrusion and epithelial remodeling taking place during the infection. This hypothesis is also supported by data showing actively extruding cell clusters (**Supplementary Figure 4**) exclusively in NTHi-infected tissues. Overall, these data suggest that bacterial clearance is functional in inverted models challenged with NTHi.

### 3.3 Mcat and NTHi infection of airway epithelia leads to the formation of IBCs and biofilm-like structures

**Figure 4:**
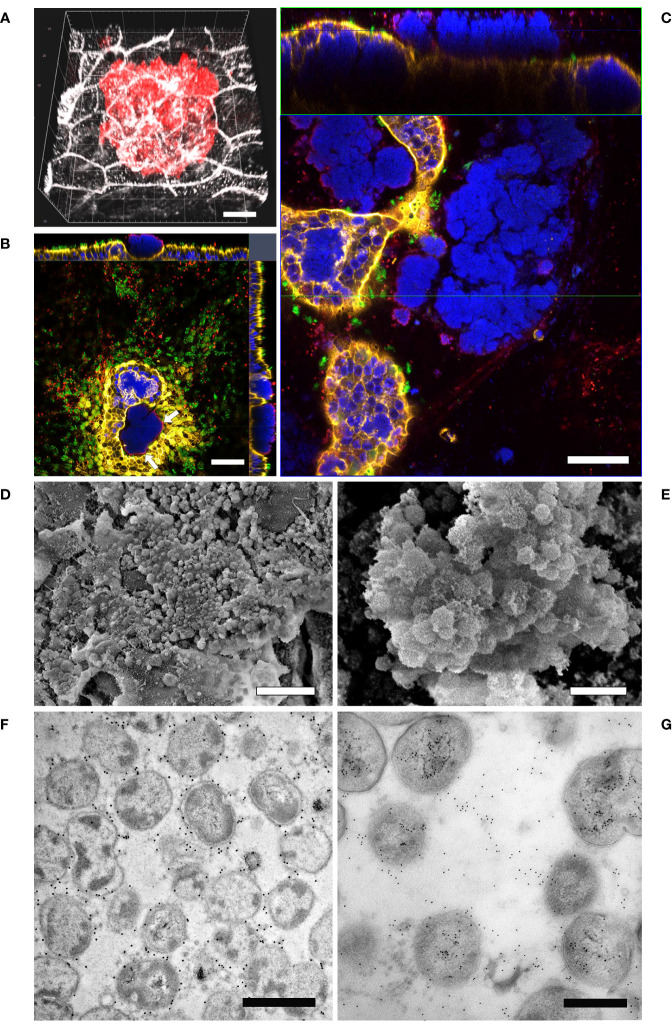
LSCM analysis of airway models infected with Mcat AERIS 415. **(A)** Mcat infection expands intraepithelially after 48 hours from the challenge (red is Mcat; grey is cell membrane; white bar = 5 µm). **(B)** Mcat colonies emerge apically from the epithelium. Only bacteria residing on the external perimeter of the aggregate (white arrows) can be efficiently immuno-stained with anti-UspA2 antibodies; white bar = 50 µm. **(C)** At seven days post infection, Mcat macroaggregates pervade the epithelial surface and most cells contain IBCs; the upper panel represent a Z/Y section of the epithelium while the lower panel is a X/Y view of the same stack (blue is DNA; yellow is F-actin; green are cilia; red is Mcat; white bar = 50 µm). **(D, E)** Scanning Electron Microscopy analysis of inverted airway models 14 days after Mcat inoculum shows the presence of an extra-polymeric substance enveloping bacterial clusters (white bar is 10 µm and 2 µm for **(D, E)** respectively). **(F)** Immunogold labeling assay conducted on Mcat biofilm ultrathin sections shows that UspA2 is expressed by bacterial cells residing inside the aggregate; white bar = 1 µm **(G)** dsDNA immunogold staining of a biofilm section; white bar = 0.5 µm.

**Figure 5:**
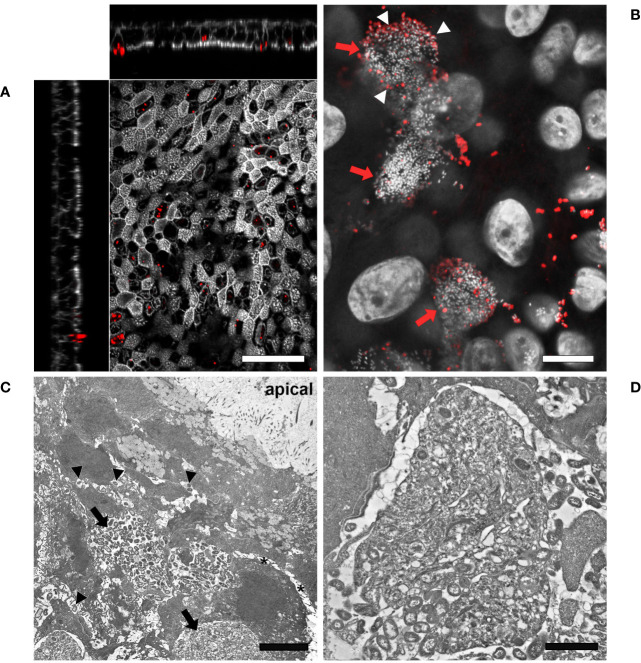
LSCM and TEM analysis of airway models infected with NTHi Fi176. **(A)** After 72 hours of infection with strain Fi176 bacteria can be found adhering to the epithelial surface, entering or inside cells; white bar = 50 µm. **(B)** NTHi forms IBCs after 1 week of infection (red arrows). Bacteria residing in the internal part of IBCs are negative for an anti-Fi176 antibody unlike those located in the margin (stained in red and indicated by white arrowheads); white bar = 10 µm. **(C)** TEM analysis of IBCs residing below the apical surface of the epithelium (black arrows). Bacteria localized paracellularly are indicated by black arrowheads. Cell-cell junctions appear loose (black asterisks); white bar = 5 µm. **(D)** Higher magnification of an IBC formed by NTHi; white bar = 2 µm.

Given the evidence that inverted models showed a lower susceptibility to bacterial infection, we decided to monitor the interaction of Mcat and NTHi with the respiratory epithelium for a longer time frame in order to identify colonization phenotypes related to bacterial persistence. In particular, we analyzed samples infected with either Mcat AERIS 415 or NTHi Fi176 strains for two weeks by using LSCM and electron microscopy (**Figures 4**, **5**). The analysis showed that, after an initial stage of adhesion, Mcat invades epithelial cells and grows intracellularly leading to the formation of IBCs (**Supplementary Figure 5**). As the infection progresses, bacteria initially colonize neighboring cells remaining confined inside the tissue (**Figure 4A**) and successively emerge from the tissue (**Figure 4B**). Once released from the epithelium, Mcat then establishes large biofilm-like structures that cover large part of the epithelium (**Figure 4C**; **Supplementary Movie 2**). During the whole process, Mcat infection is associated with macroscopical actin remodeling (**Supplementary Figure 6**). Interestingly, although samples were extensively permeabilized, only bacteria residing in the peripheral zone of aggregates could be efficiently immuno-stained with an anti-UspA2 antiserum, a protein known to be surface exposed in Mcat (Blakeway et al., 2017). This selective labelling led us to hypothesize that either UspA2 protein could be differentially expressed depending on the position of bacteria in aggregates or that antibodies could not penetrate this dense structure formed by Mcat. To verify this second possibility, we performed immunogold labelling of infected samples since the procedure would have exposed the internal core of bacterial aggregates, allowing to detect the UspA2 antigen if present. As shown in **Figure 4F**, a specific signal for UspA2 was detected also inside bacterial aggregates indicating that immunoglobulins cannot penetrate deeper into the aggregate. Moreover, SEM analysis shows the presence of an extra-polymeric substance enveloping bacterial clusters (**Figures 4D, E**), supporting the assumption that these macro-aggregates constitute, actually, a bacterial biofilm. In line with this hypothesis, extracellular DNA (eDNA) which is a structural constituent of bacterial biofilms (Goodman and Bakaletz, 2022) was also found in bacterial aggregates by immunogold analysis targeting double-stranded DNA (**Figure 4G**). Microscopy analysis conducted on samples infected with NTHi Fi176 strain revealed that, at early infection stages, NTHi is present as a single organism adhering to the cell surface or also intracellular (**Figure 5A**). After one week of infection, we observed that NTHi Fi176 was capable to form IBCs that, similarly to those formed by Mcat, were recalcitrant to antibody staining (**Figure 5B**; **Supplementary Figure 7**). Differently from Mcat that with the progress of infection formed extracellular biofilm-like structures, NTHi Fi176 strain rarely formed large colonies on the epithelium and tended to remain in single cell or small aggregate state. This evidence, in accordance with other studies, suggests that both Mcat and NTHi can adopt an intracellular lifestyle during colonization of the human airways, likely to avoid host clearance and persist within the tissue.

## 4 Discussion

The present work aims at implementing relevant cellular models for the characterization of infections caused by non-typeable *Haemophilus influenzae* and *Moraxella catarrhalis*, pathogens frequently associated with AECOPD, otitis media and other human diseases. Cellular models have been extensively used to study many biological processes including bacterial pathogenesis; however, differences between *in vitro* and *in vivo* conditions can affect the accuracy of the derived observations. In this context, advanced methods and technologies provide valuable alternatives to traditional *in vitro* systems. Organotypic airway tissue models grown at the Air-Liquid-Interface contain multiple features of the human respiratory mucosa (presence of multiple cell types, mucus production, ciliary beating, regenerative capability) providing a physiological *in vivo*-like environment that is relevant for the investigation of the host-pathogen interaction.

Nevertheless, despite the fact that traditional ALI models exhibit natural clearance mechanisms, pathogens cannot be efficiently removed from the tissue during the infection due to spatial confinement. This aspect becomes of particular importance when modelling persistent infections since, in general, cellular *in vitro* systems do not tolerate bacterial presence for a prolonged period. Hence, we hypothesized that an alternate configuration of the ALI model, based on the inversion of the epithelium, would allow one to exploit natural clearance systems to limit bacterial infection. Consistent with this hypothesis, we found that traditional ALI systems infected with Mcat lose the integrity of the epithelial barrier already after 48 hours and the tissue becomes completely saturated by bacteria within 3 days while, on the contrary, inverted models preserve their barrier integrity and display a limited number of infecting bacteria at the same time points. This evidence is also supported by the higher cytotoxicity measured in standard models at 3 days post-infection. Likewise, when comparing standard and inverted models infected with a strain of NTHi, the number of bacteria found on standard models was significantly higher at both early and late timepoints and the barrier integrity was compromised already at 2 days post-infection even though it partially recovered after 8 days. Contrary to what was observed during Mcat infection, the cytotoxicity of standard models infected with NTHi for 3 days was comparable to that of inverted samples and uninfected controls possibly because the infection with the strain Fi176 induces limited cytotoxicity at these timepoints. Interestingly, inverted samples infected with Fi176 for longer periods display a thinner epithelium and a lower number of ciliated cells respect to standard cultures suggesting that, during the course of infection, the epithelium underwent extensive remodeling probably caused by the active extrusion of invaded cells. Altogether these results suggest that during infection of inverted epithelia the shedding of infected cells and MCC activity could functionally remove bacteria from the tissue even though we cannot exclude the contribution of other factors (*e.g.* gravity). Still, this makes possible to monitor the host-pathogen interaction for longer periods and spotlight bacterial mechanisms associated with survival and persistence within the host. In this context, our results confirm that both NTHi and Mcat strains are capable of invading respiratory cells and forming IBCs *in vitro*. This intracellular form of bacterial resistance, first described for uropathogenic *E. coli*, provides access to nutrients and protection against antibiotics and innate immune effectors (Anderson et al., 2004; Justice et al., 2004). The IBC formation is pivotal for acute urinary tract infection, resulting in a latent intracellular population that acts as a reservoir for future infection. The evidence that NTHi and Mcat IBCs have been found in samples isolated from humans strongly supports the hypothesis that this form of resistance could be also crucial for the persistence of these pathogens during infection of human respiratory mucosa. Our *in vitro* data suggest that Mcat invades respiratory epithelial cells after a few hours and forms IBCs that subsequently expand to neighboring cells before emerging from the tissue and forming biofilm-like structures that rapidly cover the epithelial surface. For NTHi the process of IBC formation is significantly slower given that these structures appear visible only after several days from the infection. The fact that we did not observe the formation of macroaggregates by NTHi Fi176 strain during the infection of respiratory models was somehow surprising since this strain is capable to form abiotic biofilm in submerged conditions (internal data, not shown). One explanation could be that in a physiological air-liquid environment where nutrients are scarce compared to liquid culture conditions NTHi growth and ability to form organized biofilm-like structures is limited. On the other hand, the fact that Mcat AERIS 415 strain shows a sustained capability to form biofilm-related structures when infecting respiratory models suggest that this pathogen could make extensive use of intra- and extracellular biofilms to survive innate clearance mechanisms and establish persistence. In conclusion this work shows that is possible to prolong the host-pathogen interaction window and bring to light complex bacterial strategies used to survive to innate immune defenses. Moreover, with the use of inverted respiratory models, it would be possible to evaluate interventional strategies focused on the prevention/reduction of IBCs and biofilm formation as an approach to fight both acute and chronic disease setting.

## Data availability statement

The raw data supporting the conclusions of this article will be made available by the authors, without undue reservation.

## Ethics statement

Ethical approval was not required for the studies on humans in accordance with the local legislation and institutional requirements because only commercially available established cell lines were used.

## Author contributions

AA: Formal analysis, Investigation, Validation, Writing – original draft, Writing – review & editing. MC: Investigation, Writing – review & editing. MDF: Investigation, Writing – review & editing. ST: Formal analysis, Investigation, Writing – review & editing. AT: Investigation, Writing – review & editing. KB: Investigation, Writing – review & editing. ID: Project administration, Resources, Writing – review & editing. SP: Project administration, Writing – review & editing. AP: Conceptualization, Supervision, Writing – original draft, Writing – review & editing.
