# Supplementary material for: Modeling airway persistent infection of Moraxella catarrhalis and nontypeable Haemophilus influenzae by using human in vitro models
Source: Front Cell Infect Microbiol. 2024 May 1;14:1397940. doi: 10.3389/fcimb.2024.1397940 (PMC11094313; doi:10.3389/fcimb.2024.1397940)
Supplement: Supplementary file 1 [file DataSheet_1.pdf]

Supplementary Material

1 Supplementary Table 1

**Primary antibodies**	**Source**	**Identifier**
Rabbit anti-UspA2	This study	N/A
Rabbit anti-NTHi Fi176	This study	N/A
Rabbit anti-Uteroglobin	Thermo Fisher	PA5102469
Mouse anti-β-Tubulin IV	Sigma-Aldrich	T7941
Mouse anti-MUC5AC	Sigma-Aldrich	MAB2011
Mouse anti-ZO1	Thermo Fisher	339100
Mouse anti-p63	Abcam	Ab735
Mouse anti-dsDNA	Abcam	ab270732
**Secondary antibodies**		
Goat anti-mouse 488	Thermo Fisher	A11029
Goat anti-rabbit 568	Thermo Fisher	A11011
Goat anti-rabbit 488	Thermo Fisher	A11008
Goat anti-mouse 20 nm gold	BBInternational	EM.GAF10
Goat anti-rabbit 20 nm gold	BBInternational	EM.GAF20
**Dyes**		
DAPI	Thermo Fisher	D1306
Phalloidin 647	Cell Signaling	8940S
LIVE/DEAD Fixable Aqua Dead Cell Stain	Thermo Fisher	L34957

Table 1 – Antibodies and dyes used in this study

1.1 Supplementary Figures

S1

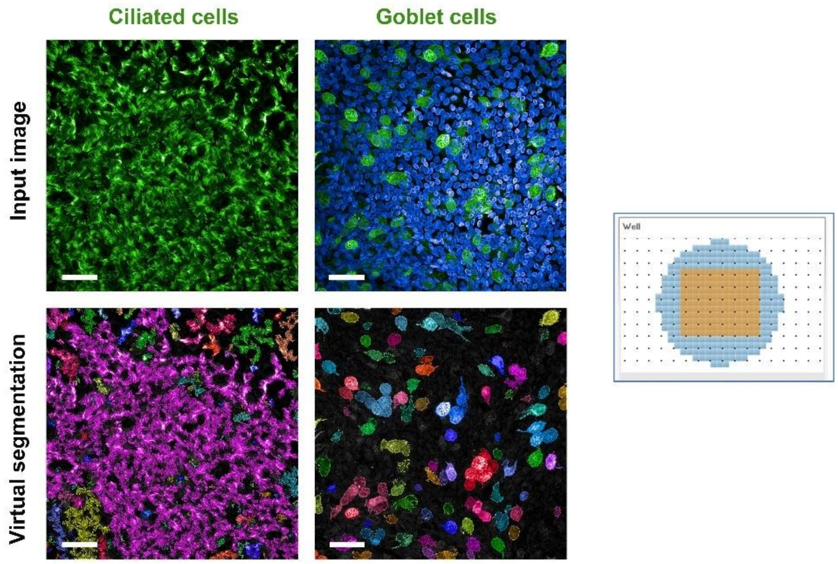

Supplementary Figure 1. Analysis of β-TUB IV and ZO-1 fluorescent signal for the quantification of cilia and goblet cells in airway models; white bars = 50 µm

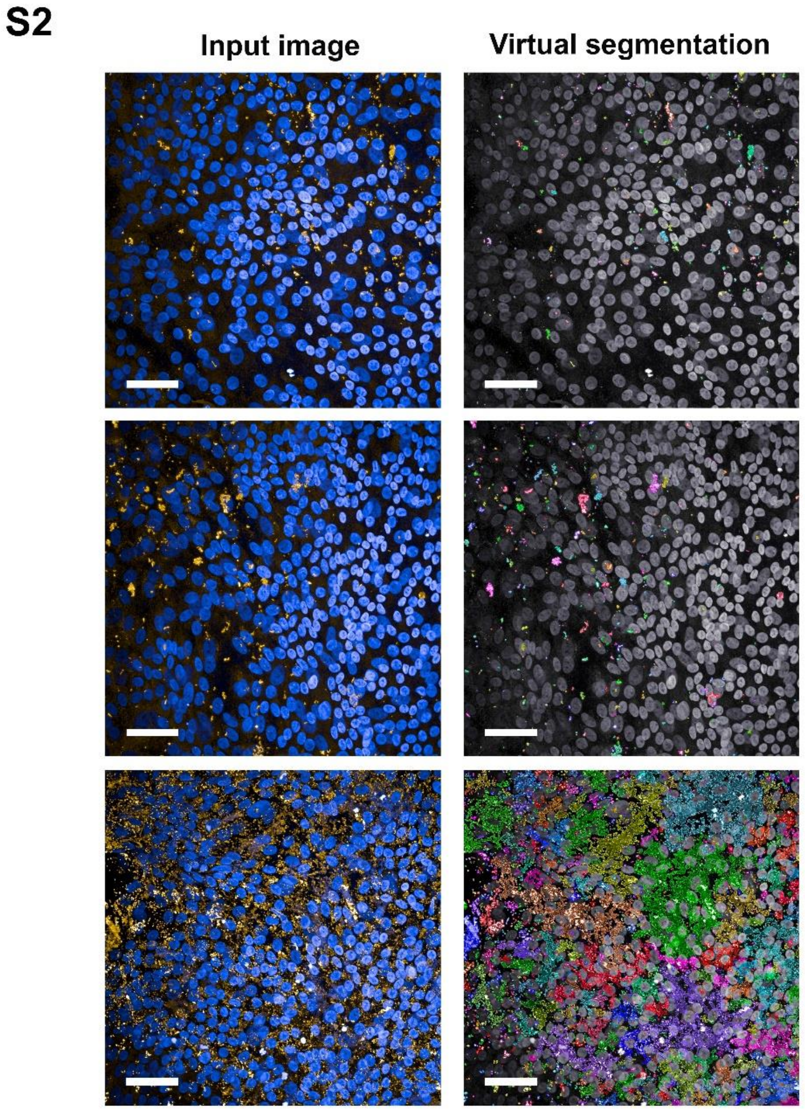

Supplementary Figure 2. Virtual segmentation of 3 different areas for quantification of NTHi Fi176 in infected airway models; white bars = 50 µm

S3

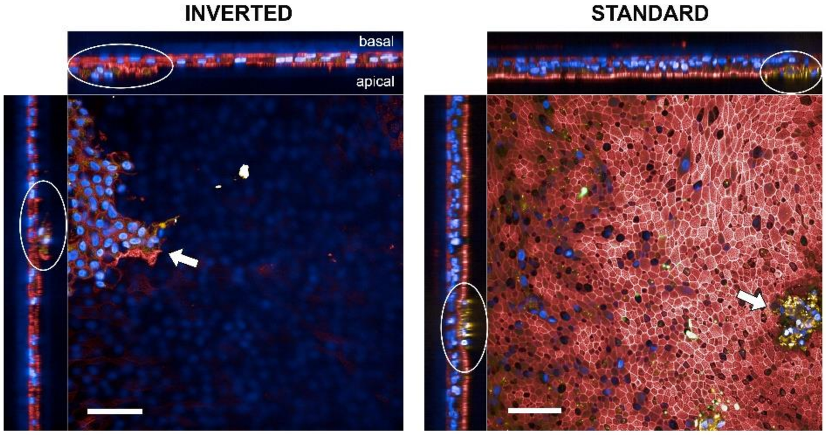

Supplementary Figure 3. Inverted models infected for 7 days with NTHi Fi176 have a reduced epithelial thickness respect to standard models. Groups of extruding cells are highlighted in both configurations (white circles). White bars = 50 µm

S4

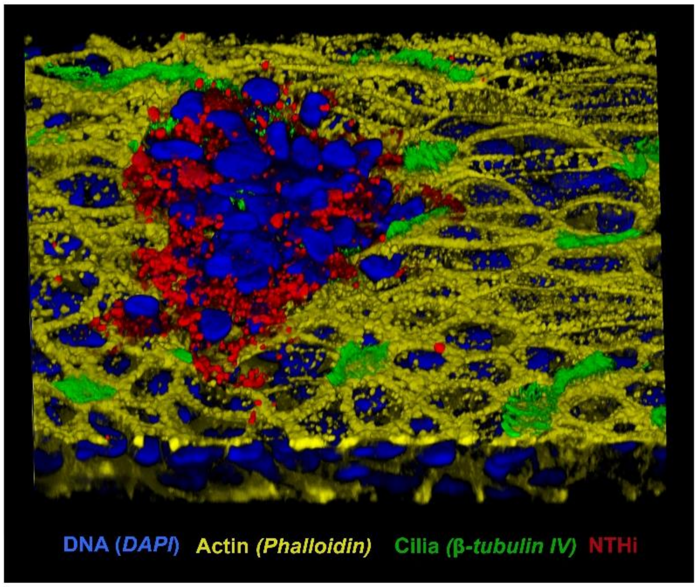

Supplementary Figure 4. 3D rendering of a confocal Z-stack showing a group of infected cells extruding from the epithelium.

S5

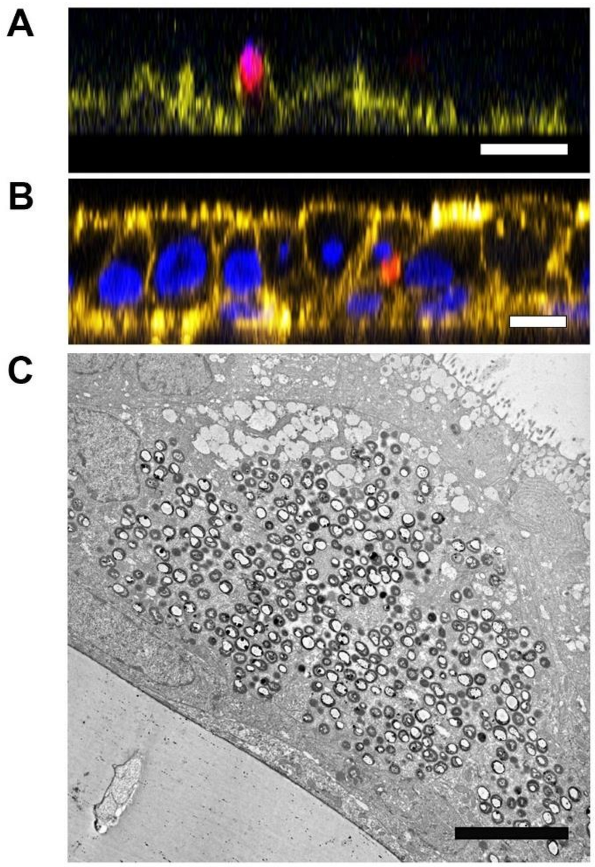

Supplementary Figure 5. A single Mcat cell entering (A) or inside (B) airway epithelial cells (yellow is F-actin, blue is DNA and red is Mcat). (C) TEM analysis of an IBC formed by Mcat AERIS 415 strain. White bars A and B = 10 µm; black bar C = 5 µm

S6

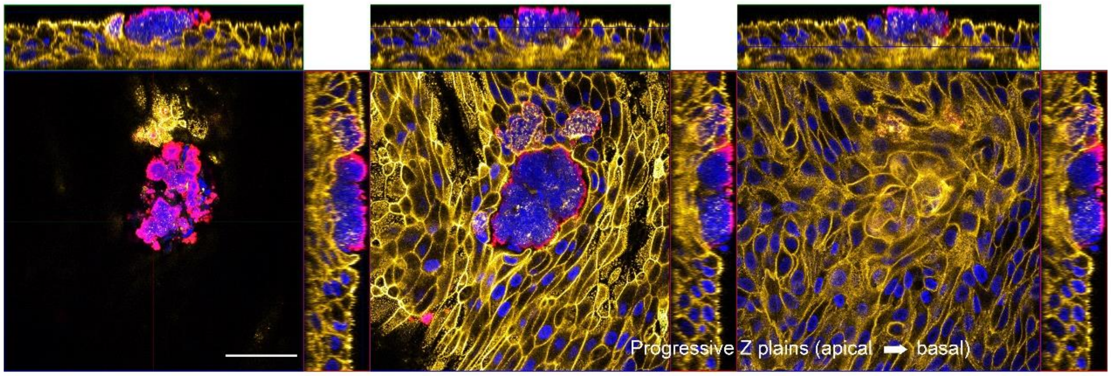

Supplementary Figure 6. Progressive orthogonal images (left to right) of an Mcat macroaggregate. Macroscopical structural rearrangement occurs in the region of contact between the colony and the epithelium. Red is Mcat, blue is DNA and yellow is F-actin. White bar = 50 µm.

S7

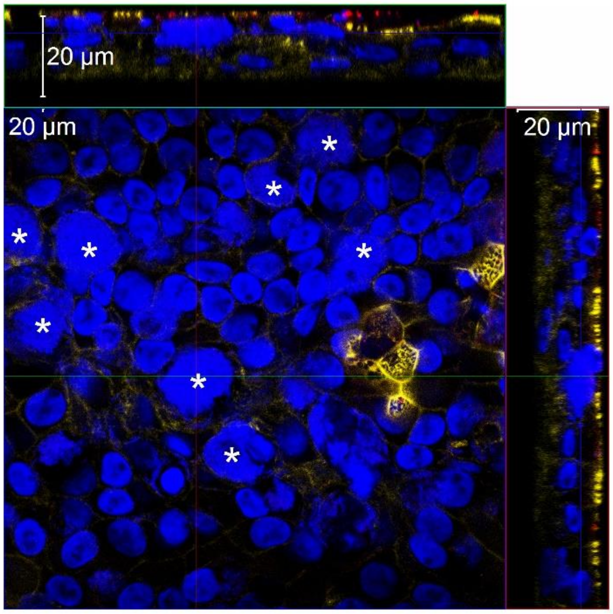

Supplementary Figure 7. Confocal analysis of a 7 days-infected airway model. Multiple IBCs formed by strain Fi176 can be identified in the deeper layers of the epithelium (white asterisks). Yellow if F-actin, blue is DNA and red is NTHi.
